# Saunders’ Pocket Medical Formulary

**Published:** 1892-04

**Authors:** 


SAUNDERS' POCKET MEDICAL FORMULARY, with an appendix containing Posological Table; Formulæ and Doses for Hypodermic Medication; Poisons and their Antidotes; Diameters of the Female Pelvis and Fetal Head; Diet List for Various Diseases; Obstetrical Table; Materials and Drugs used in Antiseptic Surgery, etc. By William N Powell, M. D., author of "Essentials of Diseases of Children," one of the associate editors of the "Annual of the Universal Medical Sciences," Chief of the Medical Clinic of the Philadelphia Polyclinic. W. B. Saunders, publisher, 913 Walnut street, Philadelphia, Pa. Price $1.75 net, buckskin; $1.50 net, cloth.

This little book contains a great number of valuable prescriptions, new and old, with blank leaves for additional formulæ by the possessor.

We can recommend it to busy practitioners, as a most valuable one. D. H. H.

